# Causal relationship between psychological factors and hepatocellular carcinoma as revealed by Mendelian randomization

**DOI:** 10.1007/s00432-024-05617-5

**Published:** 2024-02-21

**Authors:** Fengming Xu, Olaf Dirsch, Uta Dahmen

**Affiliations:** 1 Department of Infectious Diseases, The First Affiliated Hospital of Zhejiang Chinese Medical University, Hangzhou, 310006 China; 2 Else Kröner Graduate School for Medical Students “JSAM”, Jena University Hospital, 07747 Jena, Germany; 3 Institute of Pathology, Klinikum Chemnitz gGmbH, 09111 Chemnitz, Germany; 4 Experimental Transplantation Surgery, Department of General, Visceral and Vascular Surgery, Jena University Hospital, 07747 Jena, Germany

**Keywords:** Psychological distress, Leisure activity, Hepatocellular carcinoma, Risk factor, Mendelian randomization

## Abstract

**Purpose:**

The impact of psychological factors on the incidence of hepatocellular carcinoma (HCC) in humans remains unclear. Mendelian randomization (MR) study is a novel approach aimed at unbiased detection of causal effects. Therefore, we conducted a two-sample MR to determine if there is a causal relationship between psychological distress (PD), participation in leisure/social activities of religious groups (LARG), and HCC.

**Methods:**

The genetic summary data of exposures and outcome were retrieved from genome-wide association studies (GWAS). We used PD and LARG as exposures and HCC as outcome. Five MR methods were used to investigate the causal relationship between PD, LARG, and HCC. The result of inverse variance weighted (IVW) method was deemed as principal result. Besides, we performed a comprehensive sensitivity analysis to verify the robustness of the results.

**Results:**

The IVW results showed that PD [odds ratio (OR) 1.006, 95% confidence interval (CI) 1.000–1.011, *P* = 0.033] and LARG (OR 0.994, 95% CI 0.988–1.000, *P* = 0.035) were causally associated with the incidence of HCC. Sensitivity analysis did not identify any bias in the results.

**Conclusion:**

PD turned out to be a mild risk factor for HCC. In contrast, LARG is a protective factor for HCC. Therefore, it is highly recommended that people with PD are seeking positive leisure activities such as participation in formal religious social activities, which may help them reduce the risk of HCC.

**Graphical abstract:**

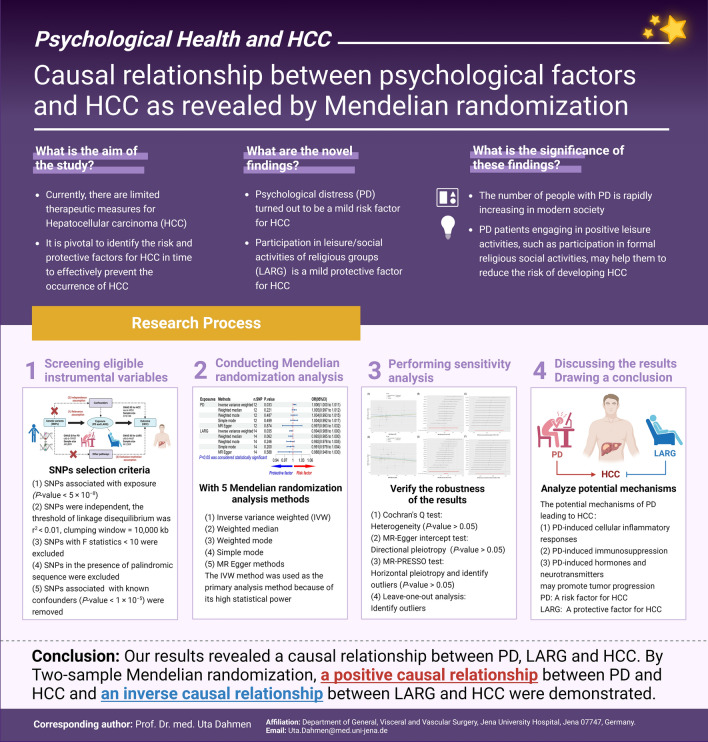

**Supplementary Information:**

The online version contains supplementary material available at 10.1007/s00432-024-05617-5.

## Introduction

Liver cancer is a major contributor to the world cancer burden. According to a 2020 Global Cancer Statistics, liver cancer ranks seventh in incidence and second in mortality among 36 common cancers (Sung et al. 2021). Hepatocellular carcinoma (HCC) is the main type of liver cancer, accounting for about 75% of all liver cancers. HCC is a serious threat to human health. HCC is characterized by insidious onset, rapid development, and easy metastasis. Therefore, diagnosis of HCC occurs mostly in an advanced or late stage of disease. Currently, there are limited therapeutic options for patients with HCC, leading to poor efficacy and short survival time (Yan et al. 2023). As a result, the incidence and mortality rates of HCC are roughly equal. The estimated global incidence of liver cancer was 9.3 per 100,000 person-years and the corresponding mortality rate was 8.5 in 2018 (Bray et al. 2018; McGlynn et al. 2021). Therefore, it is pivotal to identify potential risk factors for HCC to effectively screen this population at risk for occurrence of this fatal disease.

Up to now, Hepatitis B virus (HBV) and Hepatitis C virus (HCV) infections remain the most important risk factors for HCC. However, their importance may decline in the future as widespread neonatal vaccination against HBV and effective treatment of HBV and HCV infections are reducing the incidence of virus-associated HCC (McGlynn et al. 2021). In contrast, the importance of other lifestyle-related risk factors for HCC such as alcohol consumption, obesity, and diabetes is increasing as the economy develops and the sedentary western lifestyle spreads in the world population. These factors may replace viral hepatitis as the major risk factors for HCC in the future (Janevska et al. 2015).

However, lifestyle factors are not the only risk factors contributing to the development of disease. Modern medical research has shown that human diseases are caused by both physiological and psychological factors, and are also influenced by social factors (Rokach 2019; Wu et al. 2022). Disease perception has changed from a purely biomedical point of view to a biopsychosocial model. Pu et al. has shown in a recent rodent study that psychological distress caused by chronic unpredictable mild stress (CUMS) protocol augmented the risk of liver cancer and promoted tumorigenesis and cancer progression (Pu et al. 2022).

In modern western society, the number of patients with psychological distress (PD) is increasing due to the accelerated pace of study and work. The high pressure of competition and the heavy workload is driving patients into pessimism, irritation, anxiety, depression, nervousness, and other negative emotions, all contributing to the development of PD (Posselt et al. 2016). According to “Household, Income and Labor Dynamics in Australia (HILDA)” survey, the prevalence of very high PD did almost double from 4.8% to 7.4% within the 10-year period from 2007 to 2017 (Butterworth et al. 2020). Therefore, we raised as first hypothesis that PD might also be a crucial risk factor for developing HCC.

In contrast, participation in leisure or social activities is known to be soothing. Studies have shown that participation in religious activities improves mental health and psychosocial well-being (Chen et al. 2021). However, there is a paucity of studies regarding the relationship of participation in leisure activities and the development of malignant diseases. Therefore, we raise the second hypothesis that participation in leisure/social activities of religious groups (LARG) may help to reduce the risk of developing HCC.

Traditional observational studies are not sufficiently persuasive since they are susceptible to a large number of confounding factors and reverse causality (Sun et al. 2021). Nowadays, Mendelian randomization (MR) has emerged as a powerful method for identifying causal relationships between risk factors and diseases via genetic variants (Single nucleotide polymorphisms, SNPs) (Li et al. 2022; Sanderson et al. 2022; Sulc et al. 2022). Unlike observational studies, MR uses genetic variants randomly assigned at conception as instrumental variables (IVs) to estimate the causal effect of exposure on outcome and can reduce bias due to confounders or reverse causation (Chen et al. 2022). Therefore, this study applied two-sample MR (TSMR) analysis to identify potential causal relationship between PD, LARG, and HCC.

## Materials and methods

### Study design

**Fig. 1:**
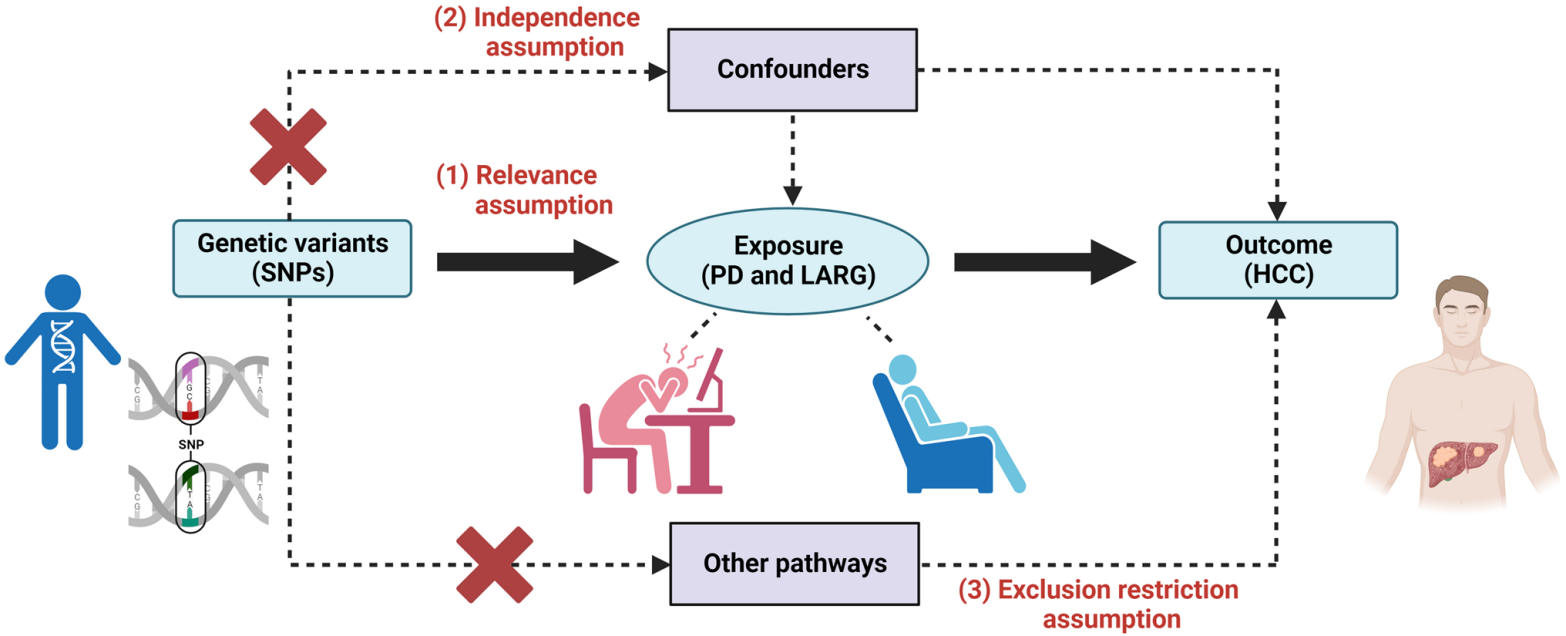
The causal relationship between PD, LARG, and HCC was explored by TSMR. The concepts and three core assumptions of the TSMR analysis are shown above. We created the figure with “BioRender.com”

In this study, we explored whether psychological health is an important factor influencing hepatocarcinogenesis by analyzing the role of two different psychological states (PD representing stress and LARG representing relaxation) in the incidence of HCC. In our analysis, PD and LARG were used as exposure factors and HCC was used as an outcome measure. There are three core assumptions that need to be met to conduct the TSMR analysis: (1) the selected SNPs should be significantly associated with exposure (PD, LARG); (2) the selected SNPs should be independent of confounders; (3) the selected SNPs are associated with the outcome (HCC) only via exposure (Fig. 1). We used summary data from publicly available databases (OpenGWAS, MRC-IEU, UK Biobank) that had obtained participant consent and ethical approval (https://gwas.mrcieu.ac.uk/).

### Data source

SNPs for PD were extracted from a genome-wide association study (GWAS) dataset (ID: ukb-b-19957) including 94,665 cases and 351,144 controls (Sample size: 445,809) of European ancestry; SNPs for LARG were extracted from a GWAS dataset (ID: ukb-b-4667) including 67,877 cases and 393,492 controls (Sample size: 461,369) of European ancestry. Summary statistic data for HCC (168 cases and 372,016 controls, Sample size: 372,184) were obtained from a GWAS dataset (ID: ieu-b-4953) of European ancestry. Specific summary information is shown in Table 1.

**Table 1:** Characteristics of data used in the Mendelian randomization study

Exposures /outcome	GWAS ID	Ethnicity	Sex	Observation sample size	Control sample size	Total sample size	Number of SNP
PD	ukb-b-19957	European	Mixed	94,665	351,144	445,809	9,851,867
LARG	ukb-b-4667	European	Mixed	67,877	393,492	461,369	9,851,867
HCC	ieu-b-4953	European	Mixed	168	372,016	372,184	6,304,034

### IVs selection criteria

**Fig. 2:**
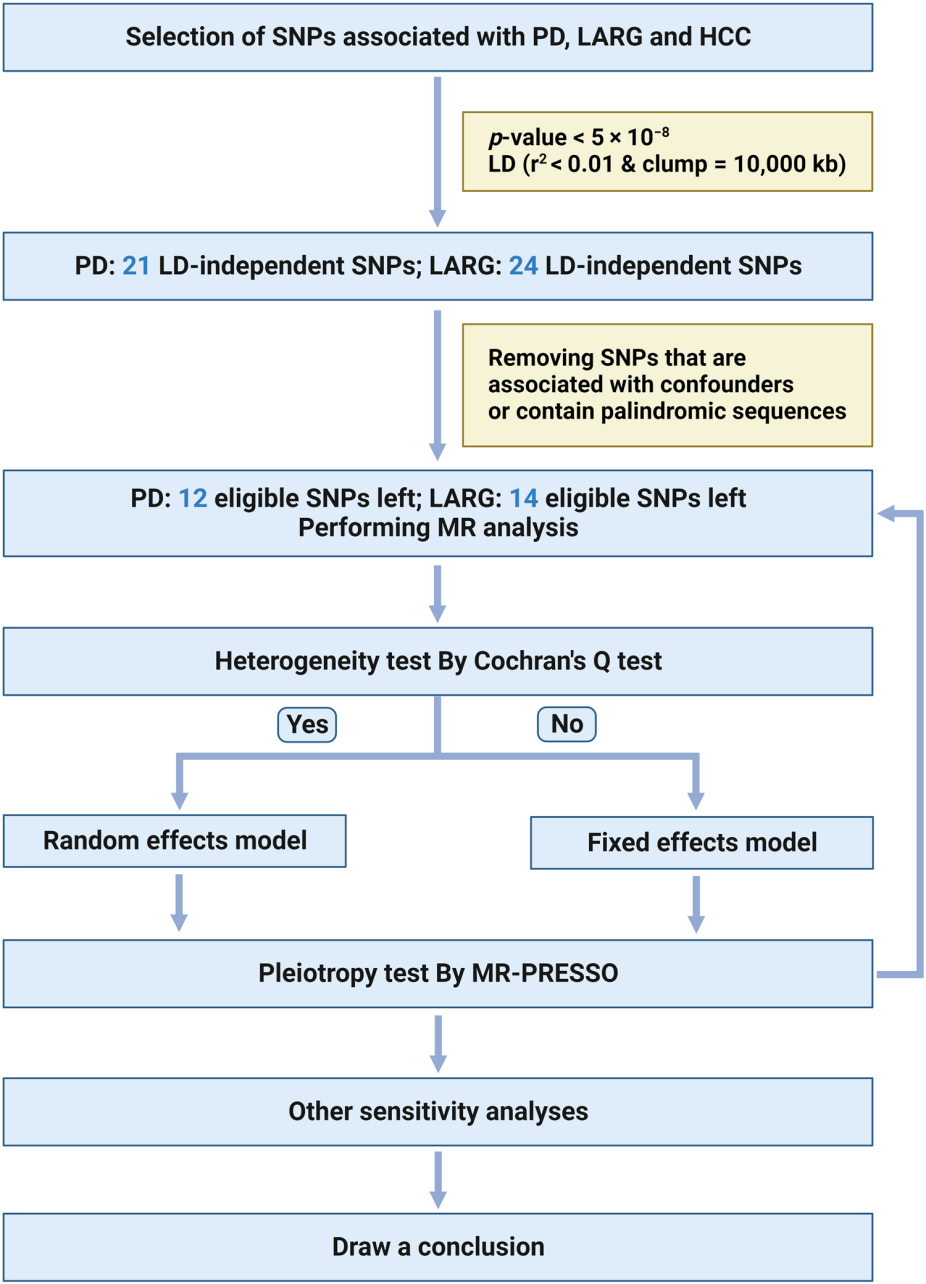
Flow diagram of the TSMR study. *MR* Mendelian randomization, *LD* linkage disequilibrium, *SNP* single nucleotide polymorphism. We created the figure with “BioRender.com.”

We selected significant and independent SNPs for exposure factors (PD, LARG) as IVs based on the following criteria:

(1) SNPs reached genome-wide association significance level of *P* < 5 × 10^−8^;

(2) SNPs were independent, and the threshold of linkage disequilibrium (LD) was r^2^ < 0.01 and clumping window = 10,000 kb;

(3) SNPs with F-statistics < 10 were excluded to avoid weak instrumental bias, and the calculation formula is $F=\left(\frac{N-K-1}{K}\right)\left(\frac{{R}^{2}}{1-{R}^{2}}\right)$; in the above formula, *R*^2^ represents the cumulative explained variance of the selected SNPs during exposure. N is the sample size of the exposure dataset, and K is the number of SNPs included in the analysis. F-statistics > 10 indicates a low likelihood of weak instrument bias;

(4) SNPs in the presence of palindromic sequence were excluded;

(5) SNPs associated (*P* < 1 × 10^−5^) with known confounders (e.g., Hepatitis A, B, C, D and E, alcohol consumption, diabetes, obesity, autoimmune hepatitis, primary biliary cirrhosis, primary sclerosing cholangitis, Wilson’s disease) were removed (Fig. 2) (Kain et al. 2022).

SNP-related phenotypes were searched via a database of human genotype–phenotype associations (http://www.phenoscanner.medschl.cam.ac.uk/).

Following the rigorous screening criteria mentioned above, 12 SNPs were used as IVs to investigate the causal relationship between PD and HCC (Table 2), while 14 SNPs were employed as IVs to study the causal relationship between LARG and HCC (Table 3).

**Table 2:** Twelve genome-wide significant SNPs were used as IVs to investigate the causal relationship between PD and HCC

SNPs	CHR*	Position	EA*	OA*	Beta	EAF	SE*	*F*-Stat*	*P* value
rs10809504	9	11,514,847	A	G	− 0.006	0.215	0.001	33.522	7.00E-09
rs12435583	14	75,207,692	A	G	0.005	0.443	0.001	39.660	3.00E-10
rs17196295	2	200,152,198	G	A	0.006	0.300	0.001	39.506	3.30E-10
rs2245310	10	93,060,349	A	G	0.005	0.318	0.001	31.007	2.60E-08
rs34478401	20	18,696,782	G	A	− 0.005	0.386	0.001	33.091	8.80E-09
rs4897473	6	131,215,271	T	C	0.005	0.358	0.001	30.023	4.30E-08
rs59970005	7	82,941,863	T	C	− 0.006	0.203	0.001	31.680	1.80E-08
rs60417486	9	98,262,178	A	G	0.009	0.089	0.002	31.252	2.30E-08
rs610877	15	59,103,328	A	C	− 0.005	0.324	0.001	33.431	7.40E-09
rs62081501	18	35,207,712	A	G	0.009	0.082	0.002	31.281	2.20E-08
rs802425	7	86,294,952	T	C	− 0.005	0.430	0.001	33.002	9.20E-09
rs9650656	8	10,607,254	G	A	0.006	0.577	0.001	50.478	1.20E-12

^*^CHR chromosome, *EA* effect allele, *OA* other allele, *EAF* effect allele frequency, *SE* standard error

**Table 3:** Fourteen genome-wide significant SNPs were used as IVs to investigate the causal relationship between LARG and HCC

SNPs	CHR	Position	EA	OA	Beta	EAF	SE*	F-Stat*	P value
rs11877152	18	42,754,468	C	T	0.007	0.113	0.001	33.879	5.90E-09
rs12119422	1	242,244,878	A	G	− 0.005	0.205	0.001	30.534	3.30E-08
rs1471093	3	108,031,094	A	G	− 0.005	0.617	0.001	52.077	5.30E-13
rs17527878	18	53,404,986	T	C	− 0.005	0.357	0.001	35.364	2.70E-09
rs332828	1	61,742,693	A	G	0.005	0.457	0.001	49.683	1.80E-12
rs34402524	4	106,196,829	G	T	0.007	0.125	0.001	39.092	4.00E-10
rs36104984	3	35,691,748	A	G	0.004	0.361	0.001	32.284	1.30E-08
rs6545977	2	63,301,164	A	G	− 0.004	0.486	0.001	31.317	2.20E-08
rs6722794	2	124,278,619	C	T	− 0.004	0.417	0.001	32.190	1.40E-08
rs6862251	5	167,558,838	T	C	− 0.005	0.302	0.001	41.032	1.50E-10
rs6944796	7	104,505,787	T	C	0.006	0.207	0.001	41.482	1.20E-10
rs699534	1	90,942,808	C	A	− 0.005	0.573	0.001	39.879	2.70E-10
rs8020432	14	34,025,195	A	C	0.004	0.699	0.001	29.806	4.80E-08
rs990702	7	97,289,021	T	C	0.005	0.240	0.001	33.614	6.70E-09

^*^CHR chromosome, *EA* effect allele, *OA* other allele, *EAF* effect allele frequency, *SE* standard error

### MR analysis

In this study, inverse variance weighted (IVW), weighted median, weighted mode, simple mode, and MR Egger methods were used to investigate the causal relationship between PD, LARG, and the outcome HCC. IVW method assumes that all SNPs are valid IVs. The IVW method was used as the primary analysis method because of its high statistical power (Zhu et al. 2023). If the *P* value of Cochran’s Q test is less than 0.05, the result of the MR analysis was analyzed using the IVW random effects model; otherwise, the fixed effects model was used.

### Sensitivity analysis

Sensitivity analysis was performed to determine the robustness of the MR results. Cochran’s Q test was used to detect heterogeneity in the data, with *P* < 0.05 indicating the presence of heterogeneity; MR-Pleiotropy RESidual Sum and Outlier (PRESSO) was used to detect horizontal pleiotropy and identify outliers. If outliers were found, they were removed and outlier-corrected MR analysis was performed to obtain unbiased causal estimate. Directional pleiotropy was assessed via the MR Egger-intercept test. The leave-one-out analysis was used to assess whether MR results were strongly driven by specific SNPs. *P* < 0.05 was deemed as suggestive significance. The MR analysis was performed using the R package TwoSampleMR (version 0.5.7). The MR-PRESSO was conducted using the R package MR-PRESSO (version 1.0) in R program (version 4.3.1).

## Results

### PD had a positive causal effect on HCC risk

**Fig. 3:**
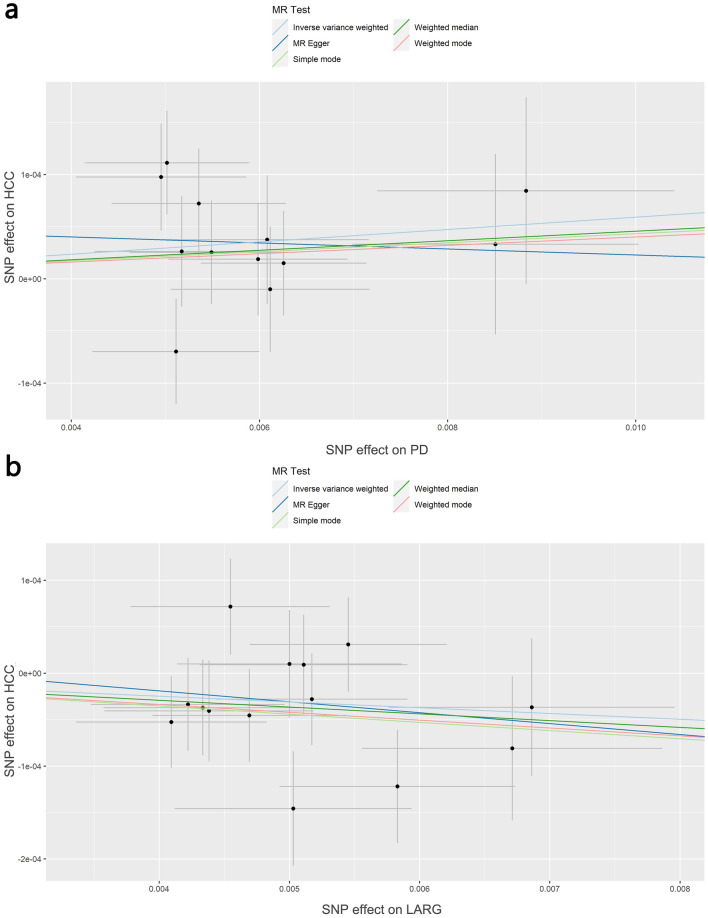
**a** Scatter plot demonstrating the causal relationship of PD on HCC; **b** scatter plot demonstrating the causal relationship of LARG on HCC

Using the 12 extracted SNPs that were eligible for IV screening (Table 2), we found a potential positive causal effect of PD on HCC incidence. In the IVW analysis, PD were significantly correlated with HCC (OR 1.006, 95% CI 1.000–1.011, *P* = 0.033). The results of weighted mode (OR 1.004, 95% CI 0.993–1.015, *P* = 0.487), weighted median (OR 1.005, 95% CI 0.997–1.012, *P* = 0.221), and simple mode (OR 1.004, 95% CI 0.992–1.017, *P* = 0.499) analyses were similar to the IVW analysis, but the results did not reach statistical significance. In contrast, the result of the MR Egger (OR 0.997, 95% CI 0.963–1.032, *P* = 0.874) analysis differed from the above analysis, but the results did not reach statistical significance as well. The estimated effect sizes of the SNPs on both PD and HCC were displayed in scatter plots (Fig. 3a).

**Fig. 4:**
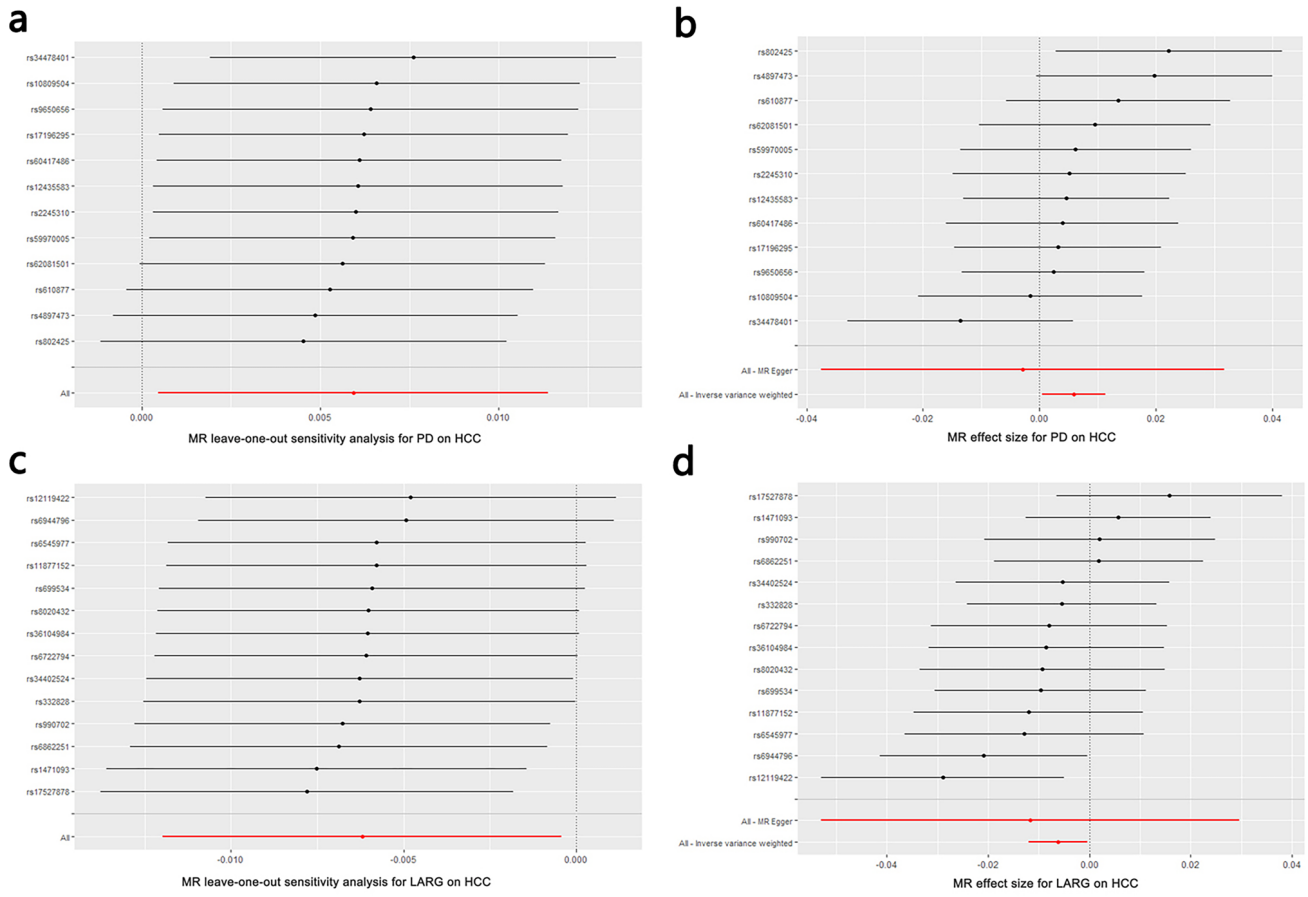
**a** Leave-one-out plot of SNPs associated with PD and HCC; **b** forest plot of SNPs associated with PD and HCC; **c** leave-one-out plot of SNPs associated with LARG and HCC; **d** forest plot of SNPs associated with LARG and HCC

We calculated the F-statistic for each SNP, and the results were all greater than 10 (ranged from 30.023–50.478, Table 2), suggesting that there is a low likelihood of weak instrumental bias. Moreover, we used Cochrane’s Q test (IVW and MR Egger methods) to detect heterogeneity in the data. The results showed no significant heterogeneity with *P* = 0.527 > 0.05 for IVW method and *P* = 0.460 > 0.05 for MR Egger method. Besides, the result of the directional pleiotropy test by the Egger-intercept method was *P* = 0.625 > 0.05, indicating that the IVs did not significantly affect the outcome through pathways other than exposure. We used MR-PRESSO to confirm the absence of horizontal pleiotropy and outliers in the data (*P* = 0.570 > 0.05) (Table 4). Furthermore, no single SNP substantially violated the generalized effect of PD on HCC incidence in the leave-one-out analysis, indicating that the results of the MR analysis were robust (Fig. 4a). The forest plot showed that PD may increase the risk of HCC (Fig. 4b).

**Table 4:** Heterogeneity and pleiotropy analyses between PD, LARG, and HCC

Exposures	Outcome	Cochrane's Q				MR-PRESSO		Egger intercept	
		IVW	*P*-val^*^	MR Egger	*P* val	Outliers	*P* val	Intercept	*P* val
PD	HCC	10.034	0.527	9.780	0.460	0	0.570	5.17E-05	0.625
LARG	HCC	12.722	0.470	12.648	0.395	0	0.489	2.79E-05	0.795

^*^val value

**Fig. 5:**
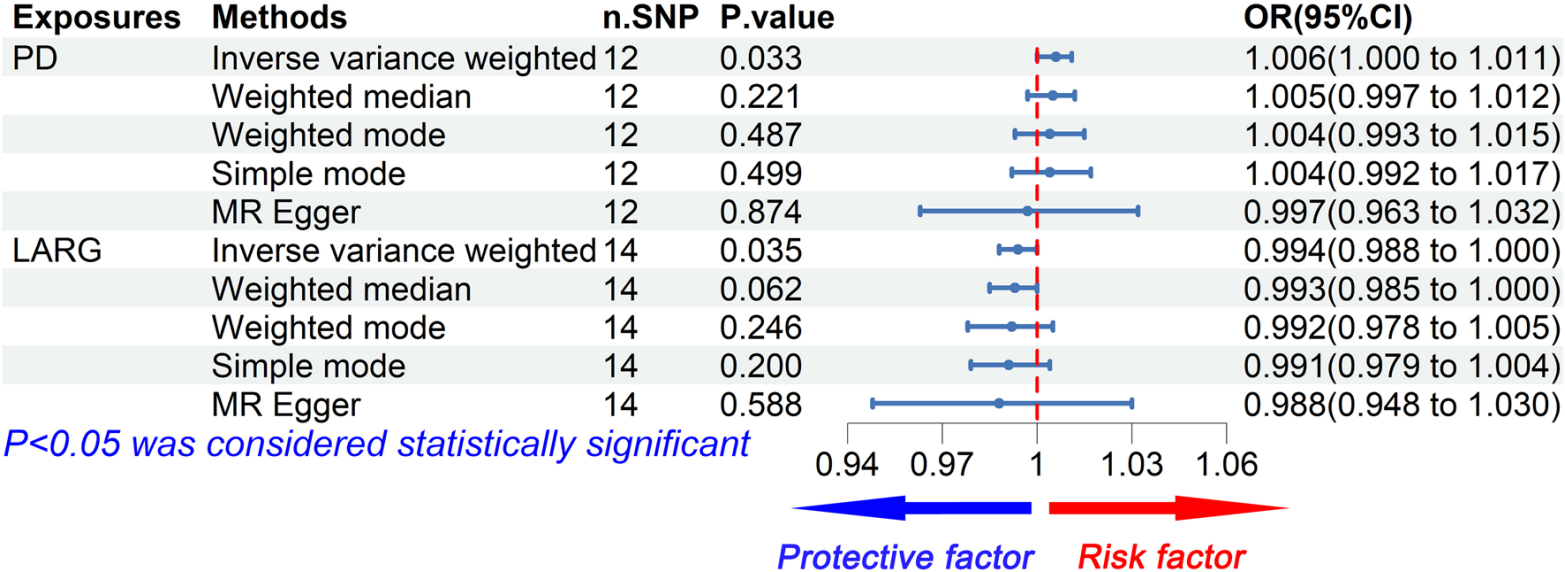
Forest plot for the causal relationship of PD, LARG on HCC. Overall, PD is a risk factor for the incidence of HCC and LARG is a protective factor for the incidence of HCC

In summary, our results revealed that PD was positively associated with the risk of HCC, suggesting that PD indeed might be risk factor, albeit a small one, for the development of HCC (Fig. 5).

### LARG had an inverse causal effect on HCC risk

Using the 14 extracted SNPs that were eligible for IVs screening (Table 3), we revealed an inverse causal effect of LARG on HCC incidence. In the IVW analysis, LARG were significantly correlated with HCC (OR 0.994, 95% CI 0.988–1.000, *P* = 0.035). The results of weighted mode (OR 0.992, 95% CI 0.978–1.005, *P* = 0.246), weighted median (OR 0.993, 95% CI 0.985–1.000, *P* = 0.062), simple mode (OR 0.991, 95% C 0.979–1.004, *P* = 0.200), and MR Egger (OR 0.988, 95% CI 0.948–1.030, *P* = 0.588) analyses were similar to the IVW analysis, but the results did not reach statistical significance. The estimated effect sizes of the SNPs on both LARG and HCC were displayed in scatter plots (Fig. 3b).

We calculated the F-statistic for each SNP, and the results were all greater than 10 (ranged from 29.806–52.077, Table 3), suggesting that there is a low likelihood of weak instrumental bias. Using Cochrane’s *Q* test (IVW and MR Egger methods) revealed the absence of heterogeneity in the data (*P* = 0.470 > 0.05 for IVW method and *P* = 0.395 > 0.05 for MR Egger method). Besides, the directional pleiotropy test by the Egger-intercept method resulted in a *P* value greater 0.05 (*P* = 0.795 > 0.05), indicating that the IVs did not significantly affect the outcome through pathways other than exposure. MR-PRESSO analysis confirmed the absence of horizontal pleiotropy and outliers as well (*P* = 0.489 > 0.05) (Table 4). Furthermore, no single SNP substantially violated the generalized effect of LARG on HCC risk in the leave-one-out analysis, indicating that the results of the MR analysis were robust (Fig. 4c). The forest plot showed that LARG may decrease the risk of HCC (Fig. 4d).

In summary, our findings demonstrated that LARG was negatively associated with HCC, suggesting that LARG might be a protective factor for HCC (Fig. 5).

## Discussion

Current research suggests that cancer development in general but also primary liver cancer is the result of the long-term combined effect of many factors. Actually the causal relationship between low-density lipoprotein cholesterol (LDL-C), telomere length (TL), rheumatoid arthritis (RA), hypothyroidism, and HCC has been shown not only in animal experiments and observational studies, but lately also using the same methodology of MR (Table 5).

**Table 5:** Using MR to evaluate potential causal factors of HCC

Author Year	Main method	Exposure	Outcome	Results
(Cao et al. 2023)	IVW	Low-density lipoprotein cholesterol (LDL-C)	HCC	LDL-C is a protective factor for HCC
(Zhang et al. 2023)	IVW	Rheumatoid arthritis (RA)	HCC	RA is a protective factor for HCC
(Yang et al. 2023)	IVW	Telomere length (TL)	HCC	No significant causal relationship was observed between TL and HCC
(Lu et al. 2022)	IVW	Hypothyroidism	HCC	Hypothyroidism is a protective factor for HCC
(Deng et al. 2022)	IVW; Wald ratio	Ever/never drinkers; alcohol consumption; coffee, tea, milk, yoghurt consumption	HCC	Ever/never drinkers and alcohol consumption are risk factors for HCC; Coffee, tea, milk, and yoghurt consumption are protective factors for HCC
(Pan et al. 2022)	IVW	Sum basophil neutrophil counts (SBNC)	HCC	SBNC is a protective factor for HCC

The role of psychological health in disease has been frequently discussed in the past. Recently the impact of psychosocial factors such as psychological health is gaining more interest. Yet relevant and reliable research evidence is scarce.

Our study is the first MR analysis to explore the role of psychological health in the pathogenesis of HCC. In this TSMR analysis, we comprehensively explored the relationship between PD, LARG, and HCC. By analyzing a large amount of sample data, our results revealed that PD is probably indeed a risk factor for HCC whereas LARG seems to be a protective factor for HCC. Although, the impact of both factors is relatively small based on the OR of 1.006 and 0.994, respectively, the results are in line with other studies using completely different approach.

Epidemiologic observational studies observed an association between psychological stress and cancer incidence (Chida et al. 2008; Cooper et al. 2023; Falcinelli et al. 2021). For example, the results of the meta-analysis by Yang et al. showed that work stress is an important risk factor for lung, colorectal, and esophageal cancers (Yang et al. 2019). The results of the case–control study by Kruk et al. suggest that psychological stress is likely to be associated with an increased risk of breast cancer (Kruk et al. 2004). However, observational studies may be subject to reverse causality, and it is difficult to avoid confounding by confounders as well.

MR refers to an analytical method for assessing the causal relationship between modifiable exposures and clinically relevant outcomes (Sekula et al. 2016). Nowadays, MR, as an emerging causal research method in epidemiology for identifying risk factors (Alzheimer’s et al. 2023), is a powerful complementary tool to enrich conventional epidemiological studies, as shown by our results.

These observational studies are further supported by basic research using animal models. Recent studies in rodents investigated the impact of psychological stressors on cancer development and the underlying molecular events.

In 2022, Pu et al. observed that depression increases the risk of HCC in rats. In their study, a rat model of HCC with depression was established by administering n-nitrosodiethylamine (DEN) and CUMS protocol (Alqurashi et al. 2022). They found that the tumor load and incidence in rats in the DEN + CUMS group were obviously higher than that of the DEN group. Furthermore, the survival rate of rats in the DEN + CUMS group was substantially lower than that of the DEN group. They further observed an epigenetic downregulation of hypocretin in the CUMS group suggesting that chronic stress might cause a molecular dysregulation facilitating tumor development (Pu et al. 2022).

Further supporting observations were reported by Hu et al. who discovered that experimentally induced depressive disorder (DD) in mice promoted HCC progression and metastasis. In their study, a mouse model of HCC with DD was established by administering DEN and Reserpine (a DD inducer). The degree of malignancy of liver tumors in the HCC-DD group of mice was higher than that in the HCC group. Further in vitro studies based on primary cell cultures showed that DD promoted tumor growth and metastasis as indicated by the enhanced migration ability in the HCC-DD group cells. This was explained by an activation of the AKT signaling pathway and upregulation of ABCG2 expression (Hu et al. 2020).

Taken together, our study revealed a small but not negligible effect of psychological distress or well-being on the development of HCC. Our findings are in line with epidemiological as well as animal studies, justifying the call for complementary preventive and therapeutic measures. Our study has several major strengths.

First, previous observational experiments in animals have usually investigated the effect of PD on HCC based on the administration of DEN to induce HCC. In contrast, our TSMR study excluded DEN to examine the causal relationship between PD, LARG, and HCC, making the research process more rigorous.

Second, it is a TSMR analysis that lends itself to causal inference. We established strict selection criteria for IVs. Only SNPs met the relevance, independence, and exclusion-restriction assumptions of the MR analysis could be selected as IVs.

Third, we used comprehensive analytic tests to detect IVs for heterogeneity and pleiotropy, and all IVs passed the heterogeneity and pleiotropy tests. Besides, we executed a series of powerful MR methods to analyze the causal relationships among PD, LARG, and HCC.

However, there are several limitations to our study:

First, not all MR analysis method results yielded valid causal relationships, which might be due to the small effect. However, vast majority of analysis methods yielded similar results. Since all IVs passed the heterogeneity and pleiotropy tests, we chose the results of the IVW method with highest test efficacy as the main reference result (Zhu et al. 2023).

Second, the source population of the dataset is European, which limits the applicability of the results to non-European populations. More research is needed in the future to validate the applicability of the results to other populations and ethnicities.

Third, the specific mechanism by which PD induces HCC remains unclear. Accumulating research evidence suggests that PD may contribute to increased tumor risk by leading to unhealthy behaviors (e.g., alcohol abuse), long-term inflammatory response, immunosuppression, and secretion of stress-related mediators (Barrett et al. 2021; Dai et al. 2020; Miller et al. 2019; Shin et al. 2016; Vignjević Petrinović et al. 2023; Wang et al. 2020). In particular, prolonged inflammatory response and the decline of the immune surveillance ability are closely related to tumorigenesis (Dai et al. 2020; Dhabhar 2014; Vignjević Petrinović et al. 2023). Further exploration of the role of PD-induced cellular inflammatory responses, immunosuppression, and stress-related mediators in HCC may help to clarify the pathogenesis of HCC.

Moreover, we attempted to further investigate the relationship between irritability and HCC, and there was no significant causal relationship between irritability and HCC (OR 1.001, *P* = 0.485. See Supplementary Information for details). We speculate that this result may be related to the release of stress during tantrums, but further research is needed to confirm this. It is worth noting that participating in a LARG is a better option for releasing stress than throwing a tantrum.

**Fig. 6:**
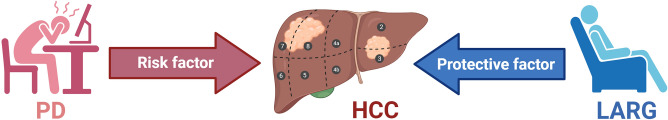
The results of TSMR showed that PD was a risk factor for HCC and LARG was a protective factor for HCC. We created the figure with “BioRender.com.”

In summary, our results revealed a causal relationship between PD, LARG, and HCC. By TSMR, a positive causal relationship between PD and HCC and an inverse causal relationship between LARG and HCC were demonstrated (Fig. 6). Although the mechanism of association between PD and HCC is not fully understood, the results of the current MR analysis suggest that psychological factors play an important role in the pathogenesis of HCC. Therefore, we suggest that the PD population should be encouraged to integrate positive leisure activities in their daily life, not only to increase their well-being, but at the same time to reduce their risk of developing HCC.

### Supplementary Information

The research data on the causal relationship between irritability and HCC are available in the supplementary information.

Below is the link to the electronic supplementary material.

Supplementary file1 (DOCX 459 KB)

## Data Availability

The data that support the findings of this study are openly available in OpenGWAS database (https://gwas.mrcieu.ac.uk/), reference number [ukb-b-19957; ukb-b-4667; ieu-b-4953].

## Abbreviations

CHR: Chromosome
CI: Confidence interval
CUMS: Chronic unpredictable mild stress
Dep: Depression
DEN: N-Nitrosodiethylamine
EA: Effect allele
EAF: Effect allele frequency
GWAS: Genome-wide association studies
HBV: Hepatitis B virus
HCC: Hepatocellular carcinoma
HCV: Hepatitis C virus
HILDA: Household, Income, and Labor Dynamics in Australia
IV: Instrumental variable
IVW: Inverse variance weighted
LARG: Leisure/social activities of religious groups
LD: Linkage disequilibrium
MR: Mendelian randomization
MR-PRESSO: MR-Pleiotropy RESidual Sum and Outlier
OR: Odds ratio
OA: Other allele
PD: Psychological distress
SE: Standard error
SNP: Single nucleotide polymorphism
TSMR: Two-sample Mendelian randomization
