# Supplementary material for: Causal relationship between psychological factors and hepatocellular carcinoma as revealed by Mendelian randomization
Source: J Cancer Res Clin Oncol. 2024 Feb 21;150(2):100. doi: 10.1007/s00432-024-05617-5 (PMC10881603; doi:10.1007/s00432-024-05617-5)
Supplement: Supplementary file 1 — Supplementary file1 (DOCX 459 KB) [file 432_2024_5617_MOESM1_ESM.docx]

**Causal relationship between psychological factors and hepatocellular carcinoma as revealed by Mendelian randomization**

**Fengming Xu ^1,2,4^, Olaf Dirsch ^3^ and Uta Dahmen ^4,^***

^1^ Department of Infectious Diseases, The First Affiliated Hospital of Zhejiang Chinese Medical University, Hangzhou, 310006 China;

^2^ Else Kröner Graduate School for Medical Students “JSAM”, Jena University Hospital, Jena, 07747 Germany;

^3^ Institute of Pathology, Klinikum Chemnitz gGmbH, Chemnitz 09111, Germany;

^4^ Experimental Transplantation Surgery, Department of General, Visceral and Vascular Surgery, Jena University Hospital, Jena 07747, Germany.

* Correspondence: Uta.Dahmen@med.uni-jena.de

**Keywords** Psychological distress; Leisure activity; Hepatocellular carcinoma; Risk factor; Mendelian randomization.

Specific summary information is shown in **Table S1**.

**Table S1** Characteristics of Irritability data used in the Mendelian randomization study

| **Exposures**  **/Outcome** | **GWAS ID** | **Ethnicity** | **Sex** | **Observation sample size** | **Control sample size** | **Total sample size** | **Number of SNP** |
| --- | --- | --- | --- | --- | --- | --- | --- |
| Irritability | ukb-a-47 | European | Mixed | 90,282 | 232,386 | 322,668 | 10,894,596 |

**Table S2** 19 genome-wide significant SNPs were used as IVs to investigate the causal relationship between Irritability and HCC

| **SNPs** | **CHR*** | **Position** | **EA*** | **OA*** | **Beta** | **EAF** | **SE*** | **F-Stat*** | **P-value** |
| --- | --- | --- | --- | --- | --- | --- | --- | --- | --- |
| rs12612050 | 2 | 122926268 | A | G | 0.006 | 0.455 | 0.001 | 30.196 | 3.91E-08 |
| rs1422192 | 5 | 87959023 | A | G | 0.010 | 0.157 | 0.002 | 38.759 | 4.80E-10 |
| rs16884419 | 8 | 89579649 | A | G | 0.009 | 0.236 | 0.001 | 43.412 | 4.44E-11 |
| rs20551 | 22 | 41548008 | G | A | 0.007 | 0.288 | 0.001 | 33.542 | 6.98E-09 |
| rs34340612 | 18 | 42232686 | A | G | -0.008 | 0.216 | 0.001 | 33.584 | 6.83E-09 |
| rs343970 | 2 | 44957915 | G | A | -0.008 | 0.188 | 0.001 | 30.537 | 3.28E-08 |
| rs35431455 | 8 | 8673736 | C | T | -0.010 | 0.467 | 0.001 | 74.635 | 5.69E-18 |
| rs3772556 | 3 | 105249211 | T | C | -0.007 | 0.706 | 0.001 | 33.690 | 6.47E-09 |
| rs4308307 | 3 | 49193216 | T | C | -0.007 | 0.622 | 0.001 | 33.929 | 5.72E-09 |
| rs4903249 | 14 | 75083881 | T | C | 0.006 | 0.488 | 0.001 | 31.650 | 1.85E-08 |
| rs58446129 | 13 | 66582410 | T | C | 0.010 | 0.146 | 0.002 | 37.173 | 1.08E-09 |
| rs6058104 | 20 | 33271052 | A | G | -0.010 | 0.824 | 0.001 | 47.437 | 5.69E-12 |
| rs7001821 | 8 | 10129773 | C | T | -0.007 | 0.617 | 0.001 | 31.602 | 1.89E-08 |
| rs7181641 | 15 | 61744669 | G | A | 0.009 | 0.148 | 0.002 | 35.451 | 2.62E-09 |
| rs7235757 | 18 | 53067954 | A | G | 0.008 | 0.311 | 0.001 | 44.852 | 2.13E-11 |
| rs776472 | 7 | 114352862 | T | C | -0.007 | 0.566 | 0.001 | 35.206 | 2.97E-09 |
| rs7805419 | 7 | 12282451 | C | T | 0.006 | 0.414 | 0.001 | 29.785 | 4.83E-08 |
| rs9390366 | 6 | 100864938 | G | T | 0.007 | 0.226 | 0.001 | 29.788 | 4.82E-08 |
| rs999483 | 9 | 135301389 | G | T | 0.008 | 0.250 | 0.001 | 35.725 | 2.27E-09 |

*Abbreviations: CHR, Chromosome; EA, Effect allele; OA, Other allele; EAF: Effect allele frequency; SE, Standard error.


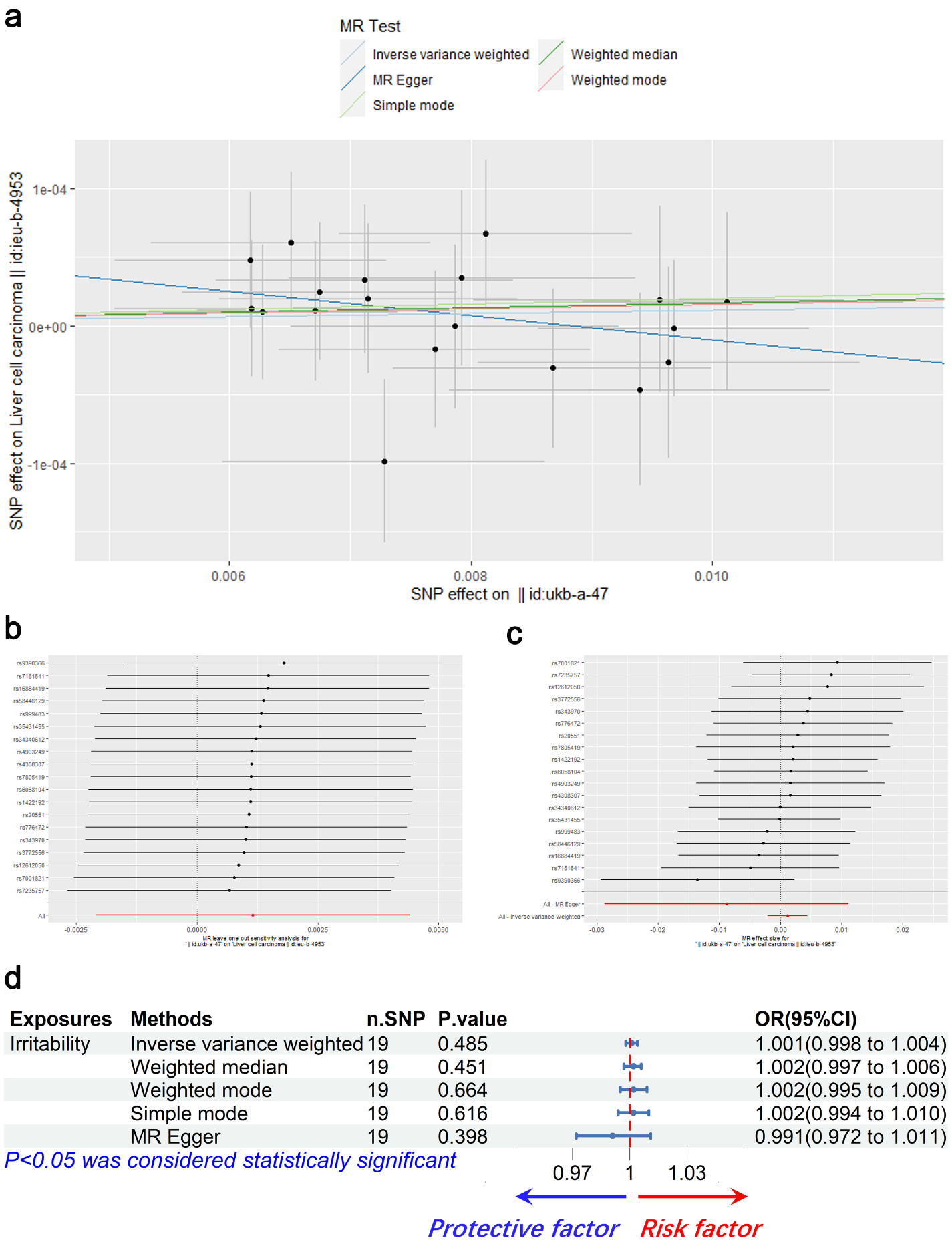


**Figure S1. (a)** Scatter plot demonstrating the causal relationship of Irritability on HCC; **(b)** Leave-one-out plot of SNPs associated with Irritability and HCC; **(c)** Forest plot of SNPs associated with Irritability and HCC; **(d)** Forest plot for the causal relationship of Irritability on HCC.

**Table** **S3** Heterogeneity and pleiotropy analyses between Irritability and HCC

| **Exposures** | **Outcome** | **Cochrane's Q** | | | | **MR-PRESSO** | | **Egger Intercept** | |
| --- | --- | --- | --- | --- | --- | --- | --- | --- | --- |
|  |  | IVW | *P*-val^*^ | MR Egger | *P*-val | Outliers | *P*-val | Intercept | *P*-val |
| Irritability | HCC | 8.559 | 0.969 | 7.571 | 0.975 | 0 | 0.972 | 7.80E-05 | 0.334 |

*Abbreviation: val, value.
